# Mesial temporal lobe epilepsy with psychiatric comorbidities: a place for differential neuroinflammatory interplay

**DOI:** 10.1186/s12974-015-0266-z

**Published:** 2015-02-25

**Authors:** Ludmyla Kandratavicius, Jose Eduardo Peixoto-Santos, Mariana Raquel Monteiro, Renata Caldo Scandiuzzi, Carlos Gilberto Carlotti, Joao Alberto Assirati, Jaime Eduardo Hallak, Joao Pereira Leite

**Affiliations:** Department of Neurosciences and Behavior, Ribeirao Preto Medical School, University of Sao Paulo (USP), Av Bandeirantes 3900, CEP 14049-900 Ribeirao Preto, SP Brazil; Center for Interdisciplinary Research on Applied Neurosciences (NAPNA), USP Ribeirao Preto, Brazil; Department of Surgery, Ribeirao Preto Medical School, USP Ribeirao Preto, Brazil; National Institute of Science and Technology in Translational Medicine (INCT-TM - CNPq), Ribeirao Preto, Brazil

**Keywords:** Temporal lobe epilepsy, Psychosis, Major depression, Astrocytes, Microglia, Aquaporin 4, Metallothionein

## Abstract

**Background:**

Despite the strong association between epilepsy and psychiatric comorbidities, few biological substrates are currently described. We have previously reported neuropathological alterations in mesial temporal lobe epilepsy (MTLE) patients with major depression and psychosis that suggest a morphological and neurochemical basis for psychopathological symptoms. Neuroinflammatory-related structures and molecules might be part of the altered neurochemical milieu underlying the association between epilepsy and psychiatric comorbidities, and such features have not been previously investigated in humans.

**Methods:**

MTLE hippocampi of subjects without psychiatric history (MTLE_W_), MTLE + major depression (MTLE + D), and MTLE + interictal psychosis (MTLE + P) derived from epilepsy surgery and control necropsies were investigated for reactive astrocytes (glial fibrillary acidic protein (GFAP)), activated microglia (human leukocyte antigen, MHC class II (HLA-DR)), glial metallothionein-I/II (MT-I/II), and aquaporin 4 (AQP4) immunohistochemistry.

**Results:**

We found an increased GFAP immunoreactive area in the molecular layers, granule cell layer, and *cornus ammonis* region 2 (CA2) and *cornus ammonis* region 1 (CA1) of MTLE_W_ and MTLE + P, respectively, compared to MTLE + D. HLA-DR immunoreactive area was higher in *cornus ammonis* region 3 (CA3) of MTLE + P, compared to MTLE + D and MTLE_W_, and in the hilus, when compared to MTLE_W_. MTLE_W_ cases showed increased MT-I/II area in the granule cell layer and CA1, compared to MTLE + P, and in the parasubiculum, when compared to MTLE + D and MTLE + P. Differences between MTLE and control, such as astrogliosis, microgliosis, increased MT-I/II, and decreased perivascular AQP4 in the epileptogenic hippocampus, were in agreement to what is currently described in the literature.

**Conclusions:**

Neuroinflammatory-related molecules in MTLE hippocampus show a distinct pattern of expression when patients present with a comorbid psychiatric diagnosis, similar to what is found in the pure forms of schizophrenia and major depression. Future studies focusing on inflammatory characteristics of MTLE with psychiatric comorbidities might help in the design of better therapeutic strategies.

## Background

Mesial temporal lobe epilepsy (MTLE) is the most common cause of intractable epilepsy in adults and is characterized by hippocampal sclerosis, neuronal loss, gliosis, and mossy fiber sprouting [1-4]. Psychiatric comorbidities are frequent in MTLE patients, and in population-based studies, epilepsy has been consistently associated with increased risk of schizophrenia [5]. However, the exact biological substrate behind the association of MTLE and psychiatric comorbidities is unknown [6,7]. We have recently shown neuropathological alterations in the hippocampus of patients with epilepsy and the history of major depression or interictal psychosis, which may indicate that structural changes and neurochemical dysfunctions may underlie psychiatric symptoms in MTLE [8-10].

Neuroinflammation-related abnormalities such as glial pathology, glutamate dysregulation, and blood-brain-barrier dysfunction are found not only in epilepsy, but also in schizophrenia and major depression [11]. Glial proteins, such as metallothionein I and II (MT-I/II), are able to quench free zinc and modulate glutamatergic neurotransmission [12], and aquaporin 4 (AQP4), found in astrocytic endfeets, is a regulator of water homeostasis that majorly controls edema formation and tissue excitability [13,14]. In schizophrenia, upregulation of MT-I/II and of astrocyte and microglia markers have been documented in several brain regions [15-17]. By contrast, neuropathological studies in specimens from major depression patients indicate reduction in hippocampal glial fibrillary acidic protein (GFAP)-positive astrocytes and of AQP4 and MT-I/II in the frontal cortex [18,19]. Protein expression and neuropathological features in MTLE with psychiatric comorbidities may resemble what is found in the pure form of the correspondent psychiatric illness [20]. Therefore, we hypothesized that expression of reactive astrocytes, activated microglia, glial MT-I/II, and AQP4 would be altered in the hippocampal formation of MTLE patients with major depression and interictal psychosis.

## Methods

### Patients

We analyzed the hippocampal formation from MTLE specimens freshly collected in the operating room and non-epileptic controls from necropsy, collected between 4 and 9 h after death. A <24-h postmortem time limit allows comparison of necropsy tissue with freshly collected surgical specimens for their protein levels, cell morphology, and tissue integrity [4,21,22]. Tissue collection and processing were conducted according to a protocol approved by our institution’s Research Ethics Board (# 2634/2008 and # 9370/2003).

MTLE specimens were derived from 43 MTLE patients who underwent a standard *en bloc* anterior temporal resection (including 3 to 4 cm of the hippocampus) for medically intractable seizures. All had clinical neuropathological confirmation of hippocampal sclerosis (HS). They were divided into three groups: 17 MTLE patients without any history of psychiatric disorder (MTLE_W_ group), 11 MTLE patients with interictal psychosis (MTLE + P group), and 15 MTLE patients with a diagnosis of major depression (MTLE + D group). For comparison purposes, 14 human non-epileptic control hippocampi from necropsies were processed and analyzed in the same manner as the surgical cases. Underlying diseases causing death were cardiomyopathy, sepsis, acute lymphoblastic leukemia, gastric adenocarcinoma, pulmonary infarct, or renal-hepatic failure, and patients had no history of hypoxic episodes during agony, seizures, or neurological diseases. Furthermore, there was no evidence of brain pathological abnormalities on clinical postmortem examination of the mesial temporal structures. MTLE and control specimens were collected between 1998 and 2008. A summary of clinical characteristics of all groups is depicted in Table 1.

**Table 1:** **Demographic and clinical data**

	**MTLE**_**W**_	**MTLE + D**	**MTLE + P**	**Controls**	***Statistics***
Male (*n*)	10	5	8	11	*No difference*
Female (*n*)	7	10	3	3	
IPI present (*n*)	7	10	8	n.a.	*No difference*
IPI absent (*n*)	10	5	3	n.a.	
Age of first seizure (years)	3.8 ± 3.3	6.3 ± 7.5	7.4 ± 8.5	n.a.	*No difference*
Age when seizures became recurrent or age of onset (years)	10.0 ± 5.4	12.0 ± 9.7	13.5 ± 7.7	n.a.	*No difference*
Seizure type: CPS (*n*)	7	9	5	n.a.	*No difference*
Seizure type: SGS (*n*)	10	6	6	n.a.	
Seizure frequency (monthly)	14.3 ± 11.4	12.6 ± 8.9	16.4 ± 11.2	n.a.	*No difference*
Right HS (*n*)	12	9	5	n.a.	*No difference*
Left HS (*n*)	5	4	5	n.a.	
Bilateral HS (*n*)	0	2	1	n.a.	
Right handedness (*n*)	14	14	11	n.a.	*No difference*
Left handedness (*n*)	2	0	0	n.a.	
Bilateral handedness (*n*)	1	1	0	n.a.	
Memory in verbal tasks: average or above (*n*)	7	7	2	n.a.	*No difference*
Memory in verbal tasks: below average (*n*)	10	8	9	n.a.	
Memory in non-verbal tasks: average or above (*n*)	11	9	3	n.a.	*No difference*
Memory in non-verbal tasks: below average (*n*)	6	6	8	n.a.	
Full-scale IQ	85.9 ± 7.7	84.9 ± 9.3	83.4 ± 7.1	n.a.	*No difference*
Years at school	7.1 ± 3.5	5.5 ± 3.5	6.3 ± 4.1	n.a.	*No difference*
Age at surgery (or at death for controls) (years)	33.5 ± 8.0	37.6 ± 11.7	40.0 ± 5.9	42.6 ± 16.0	*No difference*
Duration of epilepsy (years)	23.9 ± 8.2	24.9 ± 13.6	26.4 ± 8.6	n.a.	*No difference*
Collected side: right (*n*)	12	11	6	6	*No difference*
Collected side: left (*n*)	5	4	5	8	
Surgical outcome: complete remission (*n*)	15	10	9	n.a.	*No difference*
Surgical outcome: only auras and/or fewer seizures (*n*)	2	5	2	n.a.	

Values indicated as mean ± standard deviation when applicable. CPS: Complex partial seizure; HS: Hippocampal sclerosis; IPI: Initial precipitant injury; MTLE: mesial temporal lobe epilepsy; MTLE + D: MTLE + major depression; MTLE + P: MTLE + interictal psychosis; MTLE_W_: MTLE subjects without psychiatric history; n.a.: Not applicable; SGS: Secondarily generalized seizures.

### Clinical features of MTLE patients

All patients were referred for pre-surgical assessment due to drug-resistant seizures as defined in the literature [23]. Patients were evaluated at the Ribeirao Preto Epilepsy Surgery Program using standardized protocols approved by the institution’s Ethics Committee and a written consent form was obtained from each patient. Pre-surgical investigation at the Epilepsy Monitoring Unit included detailed clinical history, neurological examination, interictal and ictal scalp/sphenoidal electroencephalography (EEG), neuropsychology evaluation, and intracarotid amobarbital procedure (Wada test) for memory and language lateralization whenever deemed clinically necessary.

Definition of MTLE followed Engel’s criteria [24]: (I) seizure semiology consistent with MTLE, usually with epigastric/autonomic/psychic auras, followed by complex partial seizures; (II) pre-surgical investigation confirming the seizure onset zone in the temporal lobe; (III) anterior and mesial temporal interictal spikes on EEG; (IV) no lesions other than uni- or bilateral hippocampal atrophy on high-resolution magnetic resonance imaging scans (reduced hippocampal dimensions and increased T2 signal); (V) clinical histopathological examination compatible with HS; and (VI) no evidence of dual pathology identifiable by any of the assessment methods described (clinical, electrophysiology, neuroimaging, and histopathology). Exclusion criteria were as follows: (I) focal neurological abnormalities on physical examination, (II) generalized or extra-temporal EEG spikes, and (III) marked cognitive impairment indicating dysfunction beyond the temporal regions (intelligence quotient (IQ) < 69).

Information regarding antecedent of an initial precipitant injury, febrile seizures, seizure types, drug regimen, and estimated monthly seizure frequency (within the 2 years prior to surgery) were retrospectively collected from medical records for each patient. Psychiatric evaluations were conducted in all MTLE patients. Each diagnosis of major depression was independently established during the presurgical evaluation by two psychiatrists with experience in psychiatric disorders associated with epilepsy, using the guidelines of the Diagnostic and Statistical Manual of Mental Disorders, 4th edition. Once a consensus on the classification of psychotic syndromes associated with epilepsy was lacking at the time of collection, and neither DSM-IV nor ICD-10 has addressed this issue specifically (for a review, please see [20]), the diagnosis of psychosis associated with MTLE was established according to Sachdev [25], meaning that patients with interictal psychosis did not experience the following: psychotic disorder temporally associated with seizures, changes in antiepileptic medications, epileptic status, delirium, and psychosis for paradoxical normalization (for review, please see [26]). This group was defined by a prolonged psychotic state that was not related to the epileptic seizures. Typically, the psychotic states closely resemble schizophrenia, with paranoid ideas which might become systematized, ideas of influence, and auditory hallucinations often of a menacing quality. The points of difference are as follows: common religious coloring of the paranoid ideas, tendency of the affect to remain warm and appropriate, and no typical deterioration to the hebephrenic state [27]. Patients had no history of previous psychiatric disorders (prior to seizure onset) or of substance dependence at any time. Global IQ was calculated after neuropsychological tests (complete Wechsler Adult Intelligence Scale, version III (WAIS-III) or WAIS-R protocol).

### Tissue collection and immunohistochemical processing

Specimens were segmented into 1-cm blocks transversely oriented to the hippocampal long axis. Blocks were placed in buffered paraformaldehyde (Sigma, St Louis, MO, USA). After 48 to 96 h, specimens were paraffin embedded for immunohistochemistry.

Immunohistochemistry was performed with antibodies that identified immunoreactivity for reactive astrocytes (GFAP, 1:500 dilution; Dako, Glostrup, Denmark), activated microglia (human leukocyte antigen, MHC class II (HLA-DR), 1:100 dilution; Dako, Glostrup, Denmark), astroglial metallothionein I/II (MT-I/II, 1:500 dilution, Dako, Glostrup, Denmark), and perivascular aquaporin 4 (AQP4, 1:200 dilution; Santa Cruz Biotechnology, Santa Cruz, CA, USA). Antibodies specificity was verified, and immunohistochemistry was performed as described in Peixoto-Santos et al. [4]. Briefly, paraffin-embedded MTLE and control hippocampi were processed together for each antibody, with overnight incubation at room temperature, and developed simultaneously in 0.05% 3,3′-diaminobenzidine tetrahydrochloride (Pierce, Rockford, USA) and 0.01% hydrogen peroxide (Merck, Darmstadt, Germany). After sufficient colorization, reaction was halted by washing in several rinses of distilled water, dehydrated through graded ethanol to xylene (Merck, Darmstadt, Germany), and cover slipped with Krystalon (EM Science, Gibbstown, NJ, USA). Adjacent sections were hematoxilin-eosin stained (Laborclin, Pinhais, Brazil) and examined for tissue integrity. Control sections without the primary antisera did not reveal staining (data not shown).

### Semi-quantitative analysis of immunohistochemistry

MTLE and control hippocampi were compared for immunoreactivity in several hippocampal formation subfields using Lorente de No’s classification [28], which included fascia dentata granular cells and hilar neurons, as well as pyramidal cells in the *cornus ammonis* region 4 (CA4), *cornus ammonis* region 3 (CA3), *cornus ammonis* region 2 (CA2), *cornus ammonis* region 1 (CA1), prosubiculum, subiculum, parasubiculum, and entorhinal cortex layer III. Immunoreactivity were estimated in 8-μm Neu-N stained slices at × 200 magnification as previously described and well established in the literature for surgical hippocampal fragments [1,3,4,29,30].

Images of each hippocampal formation subfield from all specimens were collected and digitized with a high-resolution CCD monochrome camera attached to an Olympus microscope. Uniform luminance was maintained and checked every ten measurements using an optical density standard and a gray value scale ranging from 0 (white) to 255 (black). In brief, all digitized images were analyzed with Image J software, following the same criteria: (I) the software identifies the gray value distribution of a subfield’s digital image; (II) the immunoreactive area is selected (that is, positive stained pixels), limited to a threshold range; and (III) the threshold range is pre-settled based on control group sections, to exclude the low-intensity gray value of background staining from the analysis. For GFAP, the threshold selected allowed the quantification of positive staining present in the soma, branches, and also of the fine and characteristic astroglial meshwork. For HLA-DR, the threshold selected allowed the quantification of proteins present in the soma and branches of the immunostained cells, whereas the MT-I/II threshold allowed the quantifications of proteins in the soma and proximal branches of astrocytes. As for AQP4, we selected a higher threshold, in order to quantify only AQP4 present in the endfeets of astrocytes (that is, perivascular AQP4). A similar approach was used by our group elsewhere [3,4]. Analyses were conducted blind to hippocampal pathology and group classification.

### Data analysis

Data were analyzed using the statistical program PASW (version 18.0) and SigmaPlot (version 11.0). Groups were compared using analysis of variance (ANOVA one way, with Bonferroni *post hoc* test) or unpaired *t* test for variables with normal distribution and Kruskal-Wallis One Way Analysis of Variance on Ranks (with Dunn *post hoc* test) or Mann-Whitney Rank Sum Test for variables without normal distribution. The Fisher Exact test was applied for comparison of relative frequencies of clinical variables between groups. Statistical significance was set at *P* < 0.05 and values presented as mean ± standard deviation (SD).

## Results

### Clinical profiles

The four patient groups did not show significant differences in gender, age, or collected side (Table 1). Clinical variables such as presence of an initial precipitant injury, age of first seizure and seizure onset, seizure frequency, type and outcome, epilepsy duration, HS side, handedness, IQ, years at school, and performance in neuropsychological tests were homogeneously distributed among MTLE groups.

All epileptic patients were on antiepileptic drugs (carbamazepine, oxcarbazepine, phenobarbital, and/or phenytoin). In addition, patients were also taking benzodiazepines (MTLE_W_ group: 11 of 17; MTLE + D group: 10 of 15; MTLE + P group: 8 of 11), fluoxetine (MTLE + D group: 5 of 15), and haloperidol (MTLE + P group: 6 of 11). No differences in neuropsychological tests between patients taking or not taking benzodiazepines, fluoxetine, or haloperidol were seen. No significant influence of fluoxetine or haloperidol was seen on hippocampal GFAP, HLA-DR, or AQP4 expression. Haloperidol influence on MT-I/II expression will be described below.

### Reactive astrocytes

**Figure 1:**
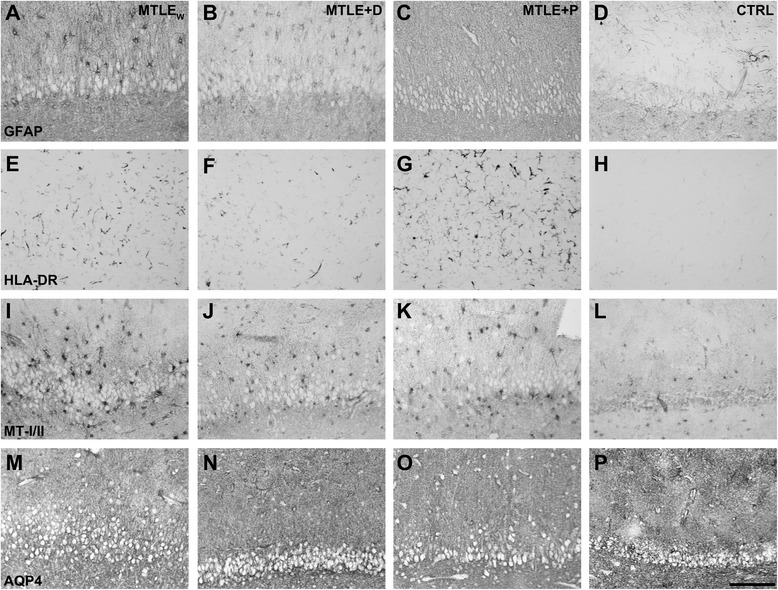
**Representative images of** ***fascia dentata*** **immunostained for GFAP (A, B, C, D), HLA-DR (E, F, G, H), MT-I/II (I, J, K, L), and AQP4 (M, N, O, P) from patients with MTLE**_**W**_ **(A, E, I, M), MTLE + D (B, F, J, N), MTLE + P (C, G, K, O) and autopsy controls (D, H, L, P).** Observe the increased astroglial reaction (A, B, C), microglial activation (E, F, G), MT-I/II immunopositive astrocytes (I, J, K), and reduced perivascular aquaporin 4 (M, N, O) in MTLE groups (MTLE_W_, MTLE + D, and MTLE + P), when compared to the respective staining pattern of the CTRL group (D, H, L, P). Bar in (P) indicates 150 μm. AQP4 = aquaporin 4; CTRL = control; GFAP = glial fibrillary acidic protein; HLA-DR = human leukocyte antigen, MHC class II; MT-I/II = metallothionein-I/II; MTLE = mesial temporal lobe epilepsy; MTLE + D = MTLE + major depression; MTLE + P = MTLE + interictal psychosis; MTLE_W_ = MTLE subjects without psychiatric history.

**Figure 2:**
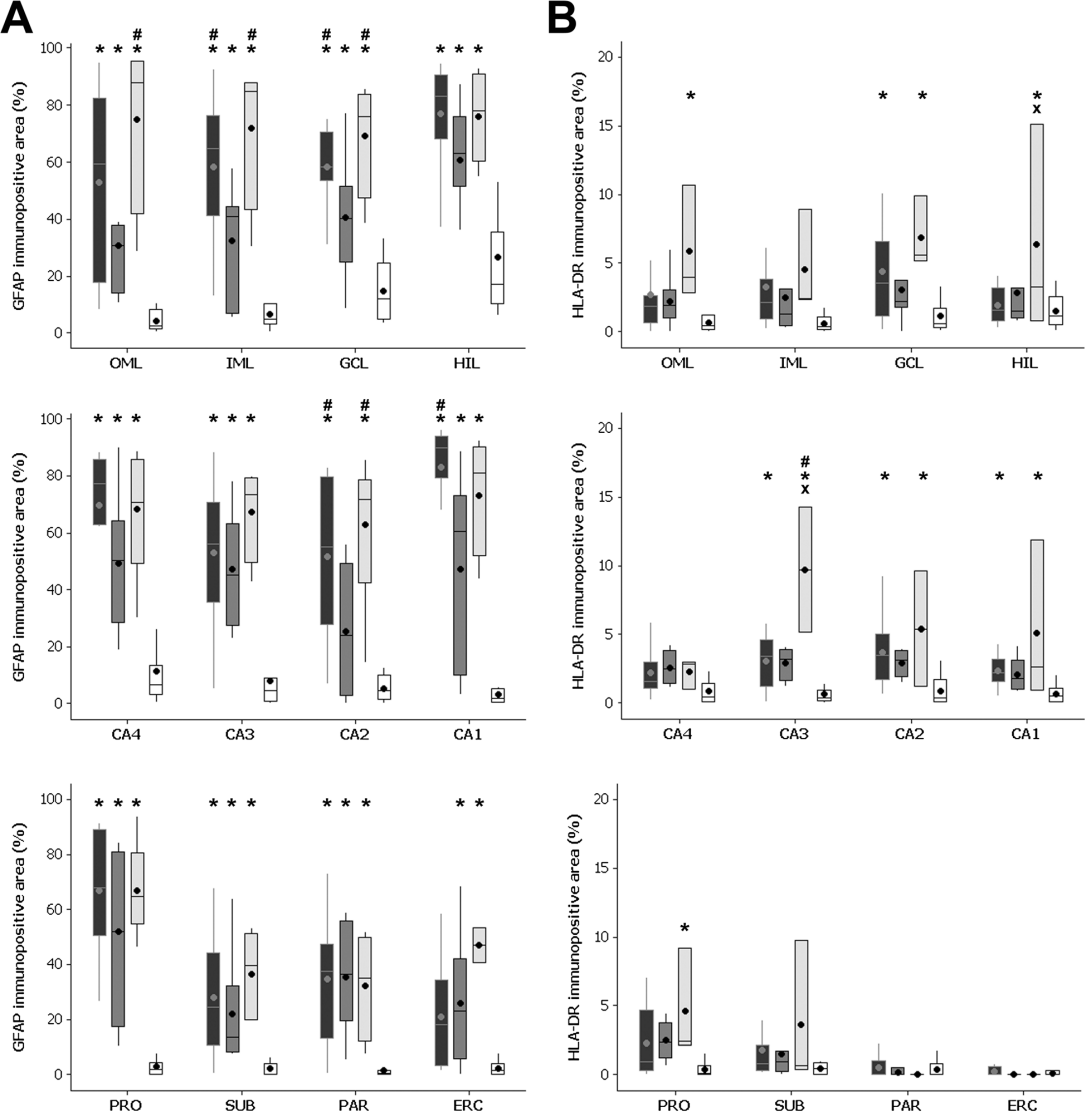
**Immunopositive area fraction of GFAP (A) and HLA-DR (B) in the hippocampal subfields of MTLE**_**W**_ **(black boxplots), MTLE + D (dark gray boxplots), MTLE + P (light gray boxplots), and CTRL group (white boxplots). (A)** All MTLE groups showed higher GFAP immunopositive area in OML, IML, GCL, HIL, CA4, CA3, CA1, PRO, SUB, and PAR (compared to CTRL). MTLE_W_ and MTLE + P had increased GFAP area in IML, GCL, and CA2 (compared to MTLE + D). MTLE + D and MTLE + P had increased GFAP area in the ERC (compared to CTRL). MTLE_W_ had increased GFAP immunopositive area in CA1, and MTLE + P had higher GFAP immunopositive area in the OML (compared to MTLE + D). **(B)** MTLE + P had increased immunopositive HLA-DR area in CA3 (when compared to all other groups), in the HIL (compared to MTLE and CTRL), and OML, GCL, CA2, CA1, and PRO (compared to CTRL). MTLE patients had increased HLA-DR immunopositive area in GCL, CA3, and CA2 (compared to CTRL). CA1 = *cornus ammonis* region 1; CA2 = *cornus ammonis* region 2; CA3 = *cornus ammonis* region 3; CA4 = *cornus ammonis* region 4; CTRL = control; ERC = entorhinal cortex; GCL = granule cell layer; GFAP = glial fibrillary acidic protein; HLA-DR = human leukocyte antigen, MHC class II; HIL = hilus; IML = inner molecular layer; MTLE = mesial temporal lobe epilepsy; MTLE + D = MTLE + major depression; MTLE + P = MTLE + interictal psychosis; MTLE_W_ = MTLE subjects without psychiatric history; OML = outer molecular layer; PAR = parasubiculum; PRO = prosubiculum; SUB = subiculum. *Indicates difference from CTRL; ^#^indicates difference from MTLE + D; and ^x^indicates difference from MTLE_W_.

Immunohistochemistry for GFAP, a marker of reactive astrocytes, showed a higher number of immunopositive cells and astrocytic processes in MTLE patients (Figure 1A,B,C), compared to staining in the controls (Figure 1D). Evaluation of the immunopositive area fraction (Figure 2A) revealed a higher GFAP area in all MTLE groups in the outer molecular layer, inner molecular layer, granule cell layer, hilus, CA4, CA3, CA1, prosubiculum, subiculum, and parasubiculum (*P* ≤ 0.024) when compared to controls. MTLE_W_ and MTLE + P had also increased immunopositive area in the inner molecular layer, granule cell layer, and CA2 (*P* ≤ 0.049) compared to MTLE + D. In CA1, MTLE_W_ had a higher immunopositive area than MTLE + D (*P* < 0.001). In the outer molecular layer, MTLE + P had a higher GFAP immunopositive area than MTLE + D (*P* = 0.013). In the entorhinal cortex, only the groups with psychiatric comorbidities (that is, MTLE + D and MTLE + P) had increased GFAP immunopositive area when compared to controls (*P* ≤ 0.032).

### Activated microglia

Activated microglia, evaluated with antibody against HLA-DR, was observed in MTLE patients as small, highly branched cells, well defined and spaced from each other (Figure 1E,F,G). In control specimens, activated microglia were rarely seen and, when present, were in much smaller number than in MTLE cases (Figure 1H). Quantitative evaluation of activated microglia (Figure 2B) revealed increased immunopositivity in the outer molecular layer, granule cell layer, hilus, CA3, CA2, CA1, and prosubiculum of MTLE + P when compared to the control (*P* ≤ 0.047). Patients of the MTLE + P group had also a higher immunopositive area in the hilus when compared to MTLE_W_ (*P* = 0.04), and in CA3 when compared to MTLE_W_ and MTLE + D (*P* ≤ 0.002). MTLE patients had an increased HLA-DR immunopositive area in the granule cell layer, CA3, CA2, and CA1 when compared to the control (*P* ≤ 0.038).

### Metallothioneins I/II

**Figure 3:**
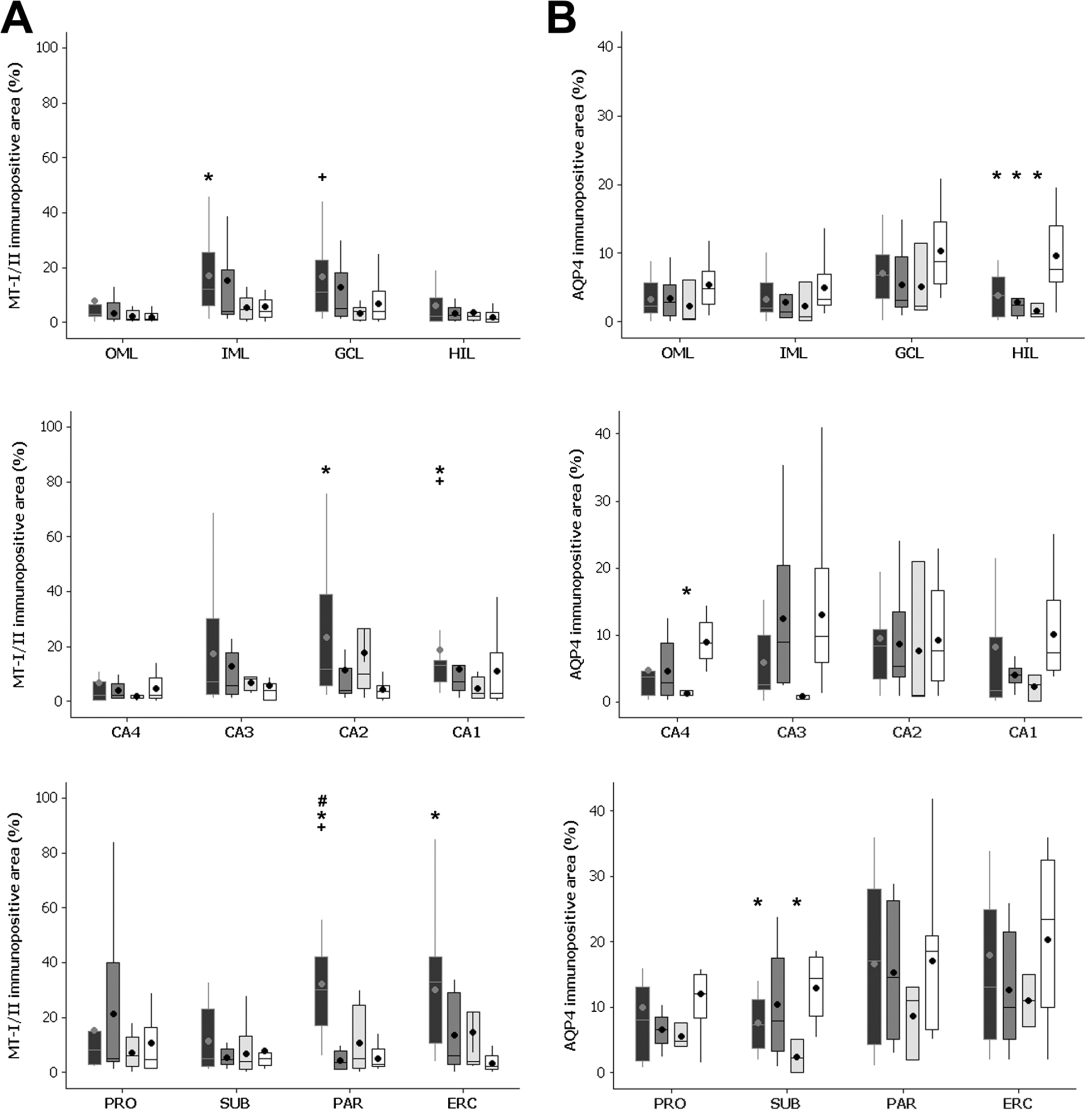
**Immunopositive area fraction of MT-I/II (A) and AQP4 (B) in the hippocampal subfields of MTLE**_**W**_ **(black boxplots), MTLE + D (dark gray boxplots), MTLE + P (light gray boxplots), and control group (white boxplots). (A)** MTLE_W_ cases had higher MT-I/II immunopositive area in the PAR (compared to all other groups), CA1 (compared to CTRL and MTLE + P), IML, CA2, ERC (compared to CTRL), and in GCL (compared to MTLE + P). **(B)** Decreased AQP4 area was observed in the hilus of all MTLE groups, in CA4 of MTLE + P, and in the SUB of MTLE_W_ and MTLE + P (compared to CTRL). AQP4 = aquaporin 4; ERC = entorhinal cortex; GCL = granule cell layer; HIL = hilus; IML = inner molecular layer; MT-I/II = metallothionein-I/II; MTLE = mesial temporal lobe epilepsy; MTLE + D = MTLE + major depression; MTLE + P = MTLE + interictal psychosis; MTLE_W_ = MTLE subjects without psychiatric history; OML = outer molecular layer; PAR = parasubiculum; PRO = prosubiculum; SUB = subiculumS. *Indicates difference from CTRL; ^#^indicates difference from MTLE + D; ^x^indicates difference from MTLE_W_; and ^+^indicates difference from MTLE + P.

The immunopositive staining for MT-I/II was observed in cells with astroglial morphology (see Figure 1I,J,K,L). Although only patients from the MTLE_W_ group had significant increase in MT-I/II immunopositive cells, qualitatively, all MTLE groups present a higher number of MT-I/II positivity in comparison to the control group (compare the micrography L with the micrographies I to K in Figure 1). Higher immunopositive staining (Figure 3A) was observed in the inner molecular layer, CA2, CA1, parasubiculum, and entorhinal cortex of MTLE_W_ when compared to the control group (*P* ≤ 0.015). Compared to MTLE + P, higher immunopositive area was observed in the granule cell layer, the CA1, and the parasubiculum of MTLE_W_ cases (*P* ≤ 0.019). In the parasubiculum, the group MTLE_W_ had a higher area fraction than the group MTLE + D (*P* < 0.001).

MT-I/II expression was increased in the inner molecular layer of the MTLE + P patients taking haloperidol when compared to those not taking it (with haloperidol, mean area fraction = 7.5 ± 3.5; without haloperidol, mean area fraction = 1.7 ± 2.2; *t* (9) = 2.885; *P* = 0.02). Also, we found a trend of increased CA2 MT-I/II expression in patients who achieved complete seizure remission after surgery (remission, mean area fraction = 20.3 ± 24.6; no-remission, mean area fraction = 9.1 ± 6.2; *t* (38) = −1.946; *P* = 0.06).

### Aquaporin 4

AQP4 immunohistochemistry revealed a reduction in the perivascular staining intensity in MTLE specimens, when compared to controls (compare micrography P with micrographies M to O in Figure 1). Compared to the control, all MTLE groups had decreased perivascular AQP4 area fraction in the hilus (*P* ≤ 0.015), whereas in CA4, only MTLE + P showed significant decrease (*P* = 0.026), and in the subiculum, MTLE_W_ and MTLE + P had reduced immunopositive area (*P* ≤ 0.015). A direct correlation was seen between IQ and AQP4 expression in the CA1 (*R* = 0.530; *P* = 0.006) and in the prosubiculum (*R* = 0.529; *P* = 0.008) of MTLE patients. Also, we found a trend to increased AQP4 expression in the CA2 of those patients who achieved complete seizure remission after surgery (remission, mean area fraction = 9.9 ± 9.0; no-remission, mean area fraction = 5.3 ± 2.9; *t* (38) = −1.930; *P* = 0.07).

## Discussion

In the present study, we investigated the expression of glial proteins GFAP, HLA-DR, MT-I/II, and perivascular AQP4 in the hippocampal formation of MTLE with and without psychiatric comorbidities and in non-epileptic controls. Comparing the MTLE groups, we found in specific hippocampal subfields an increased immunoreactive area of GFAP and HLA-DR and decreased MT-I/II and AQP4 in specimens from the MTLE patients with psychosis; while in specimens from patients with MTLE and major depression, GFAP and MT-I/II were decreased. Differences between MTLE and controls, in astrogliosis, microgliosis, increased MT-I/II, and decreased perivascular AQP4 in the epileptogenic hippocampus, were similar to what is currently described in the literature [4,31]. Given that differences between epileptogenic and control hippocampi are already well established in the literature, our discussion will focus mainly on psychiatric subgroups and their differences when compared to MTLE without psychiatric comorbidities, unless otherwise specified.

Studies in humans and animal models of epilepsy have shown upregulation of several inflammatory molecules [32,33]. However, only a few experimental studies have focused on inflammatory changes in correlates of major depression comorbid with epilepsy. For example, rats injected with pilocarpine exhibit behavioral equivalents of anhedonia and despair and alterations in inflammatory molecules as found in human major depression [34,35]. No information regarding neuroinflammatory mechanisms in psychosis of epilepsy is available to date.

Astrogliosis and microgliosis are part of a common response to injury. Although the reactive astrocyte expression profile may depend on the type of inducing injury, increased inflammatory-related molecules are always found in reactive astrocytes [36]. Interleukins 1b (IL-1beta) and 6 (IL-6) are among the molecules released after injury that can lead to glial reaction [37]. In fact, the crosstalk between activated microglia and reactive astrocytes seems crucial to the maintenance of chronic gliosis [38]. Increased astrocytic GFAP expression, a marker of reactive astrogliosis, is a common finding in the hippocampus of MTLE patients [3,4]. Likewise, we detected an increased GFAP immunoreactive area in all hippocampal subfields of MTLE patients. In patients with MTLE + D, GFAP expression levels were intermediary between the controls and MTLE_W_ or MTLE + P. Since studies have shown that patients with major depression have reduced GFAP expression [18,39], it is possible that the mechanisms underlying the decreased GFAP expression observed in major depression counterbalance the increase found in epilepsy, resulting in the intermediary values observed in our MTLE + D cases. In schizophrenia, a study showed that increased GFAP expression in the prefrontal cortex of patients with schizophrenia is increased [16], but other cortical areas and the hippocampus have shown inconclusive results [17,40,41]. In MTLE patients with interictal psychosis, we found increased GFAP expression, especially when compared to the MTLE + D cases, in agreement with a recent hypothesis that astrocyte pathology may be associated with psychotic symptoms, although the exact nature of this change remains unclear [16]. In particular, increased GFAP in schizophrenia/psychotic symptoms could be closely related to increased neuroinflammatory markers [42], as well as to increased IL-1beta and IL-6 serum levels [43]. A recent study comparing MTLE hippocampi from patients with and without *de novo* psychosis (postoperative psychosis) analyzed GFAP expression and found no qualitative differences between groups [44]. In our present series, quantitative differences in GFAP between MTLE_W_ and MTLE + P were also subtle, and major differences were seen in respect to the MTLE + D group.

Increased cortical and hippocampal HLA-DR+ microglia has been described in schizophrenia [17,45], in accordance to our findings in several hippocampal subfields of MTLE patients with interictal psychosis. Of note, increased hippocampal HLA-DR was particularly associated with paranoid schizophrenia [45], a core symptom especially represented in interictal psychosis [20]. HLA-DR levels in MTLE specimens from patients without psychiatric comorbidities and in those with major depression were similar and higher than in the controls, although statistically significant differences were detected only in the granule cell layer and CA3-1 of MTLE_W_ versus control. Microglia is an important source of inflammatory molecules [32], and a high expression of pro-inflammatory cytokines is observed in major depression [46]. Similar apparent microglial activation in MTLE_W_ and MTLE + D could partially explain why patients with epilepsy frequently develop mood disorders, an association still incompletely understood [47]. However, the levels of microglial-related inflammatory molecules such as cytokines remain to be evaluated in human epilepsy with and without major depression.

Metallothioneins are regulators of free zinc levels, an important modulator of glutamatergic neurotransmission [12]. Besides metals, oxidative stress agents and inflammatory molecules can induce MT-I/II expression [12]. Knockout mice for IL-6 have low microglial activation and low expression of MT-I/II, indicating a crucial role of inflammation in MT-I/II expression [48]. In fact, mice overexpressing MT-I/II show reduced microgliosis and reduced levels of interleukins following kainic acid *status epilepticus* [49]. In psychiatric diseases, MT-I/II gene expression in the prefrontal cortex has been found increased in schizophrenia [15] and decreased in major depression [19]. No reports are available regarding the hippocampus, but in our series, we found decreased values in cases with psychosis and in those with major depression when compared to MTLE without psychiatric comorbidities in several hippocampal subfields. Interestingly, we have found in other series of patients decreased mossy fiber sprouting in MTLE + P and increased in MTLE + D [8,9]. Since mossy fibers are zinc enriched and MT-I/II chelates zinc, cadmium, and copper, it would be expected that hippocampi from MTLE + D have a deficient metal homeostasis and likely zinc excess in neurons and glial cells and in the neuropile. A possible mechanism would be through zinc overflow from serum to brain [50] due to an inefficient blood-brain barrier in major depression [51]. In fact, low-serum zinc is a hallmark of major depressive disorders [52]. Our results of decreased hippocampal MT-I/II in MTLE associated to psychosis can be related to decreased hippocampal zinc levels/mossy fibers in interictal psychosis [8,9], as well as in schizophrenia [53,54]. Interestingly, MTLE + P patients taking haloperidol showed increased expression of MT-I/II in the inner molecular layer. In the amphetamine animal model of schizophrenia, zinc administration is able to revert behavioral equivalents of positive symptoms [55], but it is unknown if systemic zinc administration alters hippocampal zinc and/or MT-I/II expression. In fact, zinc is a ligand of the haloperidol-sensitive sigma 2 receptor in the mossy fiber of rats [56], suggesting that an increased MT-I/II expression in the mossy fiber of MTLE + P patients would facilitate zinc chelation and proper haloperidol binding. We also found a trend to increased MT-I/II in the CA2 of patients who achieved complete seizure remission after surgery, in agreement to the MT-I/II role in the control of excitability [57]. Likewise, other recent evidences indicate that hippocampal expression of proteins used as markers of full-blown epileptogenesis might be able to predict seizure outcome [58,59].

AQP4 is the main water channel in the central nervous system and presents with multifaceted functions. In inflammatory conditions, microglia can release IL-1beta, which in turn induces AQP4 expression in astrocytes [60]. AQP4 is able to regulate brain response to insults or injury, and also to influence synaptic plasticity and behavior [61]. In the AQP4 knockout mouse, memory is impaired [62]. In accordance, our results showed a direct correlation between perivascular AQP4 expression in the Sommer sector and IQ scores. AQP4 participation in synaptic plasticity and cognition occurs together with neurotrophin (NT) receptors [62]. Of note, NTs and NT receptors are differentially regulated in MTLE with psychiatric comorbidities [9,58], which could further change how AQP4 modulates plasticity. For instance, the brain-derived neurotrophic factor (BDNF) is increased in MTLE_W_ but decreased in MTLE + P [9]. In addition, tyrosine kinase receptor type 2 (TrkB) (a BDNF receptor) is increased in MTLE + P but not in MTLE_W_ [58]. Given that low levels of AQP4 associated with increased BDNF and TrkB or p75 neurotrophin receptor (p75NTR) may result in increased excitability, AQP4 levels near to control levels could be more efficient in controlling excessive excitatory activation trough a BDNF-TrkB or p75NTR loop [62,63]. In fact, we found a trend to increased AQP4 in cases with complete seizure remission, thus reinforcing the role of AQP4 in neuron activity. In addition, AQP4 has an important role in K^+^ homeostasis [13,64], and AQP4 knockout mice have higher seizure threshold but longer seizure duration [13].

## Conclusion

In summary, we described hippocampal neuroinflammatory-related molecules that show a distinct pattern of expression when MTLE patients present with a comorbid psychiatric diagnosis of interictal psychosis or major depression. Studies have reported successful treatment of patients with seizures, schizophrenia, and major depression using drugs with anti-inflammatory effect as an add-on therapy [65-68]. Given the differential expression of neuroinflammatory-related molecules in MTLE with psychiatric comorbidities, these patients could also benefit from a more targeted treatment. Further research is needed to expand and validate these findings and to better investigate possible causal mechanisms.

## Abbreviations

AQP4: aquaporin 4
BDNF: brain-derived neurotrophic factor
CA1: *cornus ammonis* region 1
CA2: *cornus ammonis* region 2
CA3: *cornus ammonis* region 3
CA4: *cornus ammonis* region 4
EEG: electroencephalogram
GFAP: glial fibrillary acidic protein
HLA-DR: human leukocyte antigen, MHC class II
HS: hippocampal sclerosis
IQ: intelligence quotient
MT-I/II: metallothionein I and II
MTLE: mesial temporal lobe epilepsy
MTLE + D: MTLE + major depression
MTLE + P: MTLE + interictal psychosis
NT: neurotrophin
p75NTR: p75 neurotrophin receptor
SD: standard deviation
TrkB: tyrosine kinase receptor, type 2
WAIS-III: Wechsler Adult Intelligence Scale, version III
